# Synergistic effects of *Bacillus subtilis* QST 713 and L-arginine supplementation during late gestation on reproductive performance in Landrace × Yorkshire sows: A randomized controlled trial

**DOI:** 10.14202/vetworld.2025.2087-2094

**Published:** 2025-07-30

**Authors:** Thepsavanh Khoudphaithoune, Do Thi Kim Lanh, Nguyen Van Thanh, Bui Van Dung, Peerapol Sukon, Nguyen Hoai Nam

**Affiliations:** 1 Department of Animal Surgery and Theriogenology, Faculty of Veterinary Medicine, Vietnam National University of Agriculture, Hanoi, 100000, Vietnam; 2 Department of Anatomy, Faculty of Veterinary Medicine, Khon Kaen University, Thailand. 123 Moo 16 Mittraphap Rd., Nai-Muang, Muang District, Khon Kaen, 40002, Thailand; 3 Research Group for Animal Health Technology, Khon Kaen University, Thailand. 123 Moo 16 Mittraphap Rd., Nai-Muang, Muang District, Khon Kaen, 40002, Thailand

**Keywords:** amino acid supplementation, *Bacillus subtilis*, L-arginine, piglet birth weight, probiotics, reproductive perf-ormance, sow nutrition

## Abstract

**Background and Aim:**

Low birth weight and within-litter variations are major challenges in swine production, often exacerbated by highly prolific sow lines. Nutritional interventions such as amino acid and probiotic supplementation have shown promise, but their combined effects remain unexplored. This study aimed to evaluate the individual and interactive effects of *Bacillus subtilis* QST 713 and L-arginine supplementation during late gestation on reproductive performance in sows.

**Materials and Methods:**

A randomized trial was conducted on 247 Landrace × Yorkshire sows allocated to four dietary groups from day 85 of gestation to farrowing: (1) Basal diet (control), (2) basal diet + *B. subtilis*, (3) basal diet + L-arginine, and (4) basal diet + both supplements. Reproductive outcomes–including individual birth weight (IBW), total litter birth weight (TBW), born-alive birth weight (NBABW), proportion of runt (<1.1 kg) and large piglets (>1.5 kg), and within-litter coefficient of variation in birth weight [CVBW])–were assessed. Linear and generalized linear mixed models were used for analysis.

**Results:**

Co-supplementation with *B. subtilis* and L-arginine significantly increased IBW (1434.7 g vs. 1310.0 g, p < 0.001), TBW (19.6 kg vs. 16.9 kg, p < 0.001), NBABW (18.1 kg vs. 15.9 kg, p = 0.006), and the proportion of large piglets (33.7% vs. 19.5%, p = 0.0002), while reducing runt piglet incidence (4.0% vs. 14.4%, p < 0.001). Neither supplement alone produced significant improvements. No treatment significantly affected litter size, CVBW, stillbirth, or mummification rates.

**Conclusion:**

Co-supplementation of sows with *B. subtilis* and L-arginine during late gestation produces synergistic impro-vements in piglet birth weight and litter quality. This strategy offers a practical and cost-effective approach to enhance swine reproductive efficiency.

## INTRODUCTION

Low birth weight piglets remain a signficant chall-enge in modern swine production due to their elevated risk of perinatal death [1], increased preweaning mortality [2], and compromised postnatal growth per-formance [3]. Moreover, marked within-litter variation in birth weight complicates herd management and can diminish the economic efficiency of pig production systems [4]. These issues have become increasingly pronounced with the widespread use of highly prolific sow lines, which, although associated with greater numbers of total and live-born piglets per litter [4], also contribute to higher rates of stillbirth, greater incidence of runt piglets, increased variation in birth weight within litters, and overall reduced birth weights [5].

Given the adverse impact of low birth weight on piglet development and survivability, various nutritional interventions have been explored to enhance fetal growth and improve related performance metrics. As the majority of fetal weight gain occurs during the final month of gestation [6], this period has been identified as a key window for nutritional modulation. One such strategy involves increasing maternal feed intake dur-ing late gestation. Although some studies have shown improved piglet birth weights when feed levels were raised from 2.5 to 3.5 kg/day during the final gestation phase [7], others have found no significant effects on litter or individual birth weights (IBWs), within-litter variation, or the proportion of piglets weighing <1 kg when sows were fed at 2.8, 3.6, or 4 kg/day [8]. A similar trend was observed in another study evaluating feed levels of 1.8, 2.3, 2.8, and 3.3 kg/day, where only the 2.3 kg level was associated with a reduced proportion of piglets under 1 kg [9]. Consequently, although increased feeding may raise production costs, it does not consistently yield improvements in piglet performance outcomes [10].

As a potentially more cost-effective alternative, the use of amino acid and probiotic supplements in sow diets has gained attention. Among amino acids, L-arginine is the most widely studied for use during late gestation, with the aim of enhancing reproductive performance. However, results on the efficacy of L-arginine supplementation have been inconsistent [11, 12]. Several studies have found no significant improvements in reproductive parameters following its use [13–16], whereas others have reported favorable outcomes, including increased IBW [17], enhanced total litter birth weight (TBW) [18], improved within-litter birth weight uniformity [19], and lower rates of stillbirth [18].

Similarly, evidence for the benefits of probiotic supplementation remains mixed [20]. While some reports suggest an increase in birth weight with probiotic use [21, 22], others have documented a decrease [23, 24]. More recently, supplementation with *Bacillus subtilis* QST 713 during late gestation has been associated with increased birth weight [25, 26] and reduced stillbirth rates [26, 27], suggesting pote-ntial reproductive benefits. Mechanistically, probiotics are thought to enhance nutrient assimilation [28], whereas L-arginine is believed to stimulate placental growth [29] and promote placental blood flow through angiogenesis [30]. These complementary mechanisms suggest that combined supplementation may have synergistic effects on reproductive performance. Nonetheless, no prior studies have investigated the interaction between L-arginine and probiotics in a fact-orial experimental design.

Despite extensive research into nutritional strategies for improving sow reproductive outcomes, challenges such as low piglet birth weight, high within-litter variations, and the incidence of runt piglets remain prevalent, particularly in highly prolific sow lines. While increased maternal feed intake during late gestation has been explored as a strategy to address these issues, the findings have been inconsistent, and its cost-effectiveness is questionable. Consequently, attention has shifted toward the use of targeted dietary supplements, such as amino acids and probi-otics, as more economical alternatives. L-arginine supplementation has been proposed to improve plac-ental development and fetal growth by enhancing angiogenesis and nutrient transport; however, studies have yielded mixed results regarding its effectiveness on reproductive parameters. Similarly, probiotics such as *B. subtilis* QST 713 have shown potential benefits in enhancing nutrient assimilation and reducing stillbirths, but the results are also inconclusive. Although both L-arginine and *B. subtilis* QST 713 have been individually studied, no previous research has investigated their interactive effects using a factorial design. Therefore, the potential synergistic benefit of co-supplementing these two bioactive agents on reproductive performance in sows remains an unexplored area.

The present study aimed to investigate the indi-vidual and combined effects of L-arginine and *B. subtilis* QST 713 supplementation during late gestation on the reproductive performance of Landrace × Yorkshire sows under commercial farming conditions. Specifically, the study sought to determine whether co-supplemen-tation would improve key reproductive parameters, including IBW, TBW, the weight of live-born piglets, and the proportions of runt and large piglets. Using a randomized factorial design, this study further aimed to clarify whether any observed effects were additive or synergistic, thereby providing critical insights into cost-effective dietary strategies for enhancing piglet quality at birth. Ultimately, the goal was to evaluate whether combined supplementation could serve as a practical intervention to optimize fetal development and reduce early-life piglet mortality in high-producing sow herds.

## MATERIALS AND METHODS

### Ethical approval

The study protocol was reviewed and approved by the Committee on Animal Research and Ethics, Faculty of Veterinary Medicine, Vietnam National Univ-ersity of Agriculture (Approval No. CARE-2023/02). All procedures adhered to national and institutional guidelines for the ethical use of animals in research.

### Study period and location

The experiment was conducted from March 2024 to January 2025 at a medium-scale commercial pig farm located in northern Vietnam (approximate coordinates: 21°16′43″N, 105°43′57″E). The region experiences a tropical monsoon climate, with hot, humid summers and cold, dry winters.

### Animals and general management

A total of 247 Landrace × Yorkshire crossbred sows (parity range: 1–7) were enrolled. From insemination to day 107 of gestation, sows were housed individually in gestation crates measuring approximately 1.2 m². One week before the expected farrowing date, they were moved to farrowing rooms and placed in similar-sized individual crates. The gestation and farrowing areas were illuminated for 10 h daily, with continuous lighting during the farrowing period. Indoor temperatures ranged from 22°C to 32°C, and humidity levels varied between 70% and 82%, maintained by fans and evaporative cooling systems–typical conditions for medium-scale commercial pig farms in northern Vietnam.

Sows were inseminated twice per estrous cycle using fresh semen from Duroc boars. From mating until day 42 of gestation, they received 1.8–2.4 kg/day of gestation feed, which increased to 2.0–2.6 kg/day until day 84. Between days 85 and 107 of gestation, the feeding level rose to 2.4–3.0 kg/day using the same gestation diet. During the last week of gestation, sows were switched to the lactation diet (2.4–3.0 kg/day), which was subsequently reduced to 1.0–1.5 kg/day during the final three days before farrowing. Feed compositions are detailed in Table 1 [31]. Water was available *ad libitum* through a bite nipple drinker system.

**Table 1:** Nutrient composition of the basal diets.

Nutrition facts	Gestation diet	Lactation diet
Crude protein (%)	13.6	16.2
Crude fiber (%)	6.2	6.0
Dry matter (%)	89.4	89.5
Crude fat (%)	7.8	6.6
Metabolizable energy (kcal/kg)	3585.4	3581.2
Total phosphorus (%)	0.77	0.8
Calcium (%)	0.95	0.91
Selenium (mg/kg)	0.65	0.69
Copper (mg/kg)	47	53
Iron (mg/kg)	235	234
Zinc (mg/kg)	279	267
Manganese (mg/kg)	235	244
Total amino acids (%)	14.2	17.4
Arginine (%)	0.94	1.21
Lysine (%)	0.99	1.29

The industrialized gestation and lactation feeds comprised corn, rice, cassava root, soybean meal, animal protein, rice bran, wheat bran, amino acids, vitamins, and minerals. The gestation diet was provided to sows during the initial 107 days of gestation. From day 108 of gestation until farrowing, sows were fed the lactation diet. Both diets meet or exceed the nutrient requirements recommended by the NRC [31] with the gestation diet focusing on maintaining optimal body condition and fetal development, and the lactation diet supporting high milk production and minimize body weight loss. Values are presented as means.

Sows were vaccinated against classical swine fever, foot-and-mouth disease, and *Escherichia coli* at gestational weeks 10, 12, and 14, respectively. Additional vaccinations for porcine reproductive and respiratory syndrome and Aujeszky’s disease were adm-inistered every 3 and 4 months, respectively. Dewo-rming was performed every 5 months, in line with the farm’s standard health management protocol.

### Treatment allocation

On gestation day 85, sows were randomly assigned to one of four dietary treatment groups using block randomization to balance parity across groups:

- Control group (n = 86): Received only the basal diet.
- *B. subtilis* group (n = 53): Basal diet + 10^10^ colony forming unit (CFU) of *B. subtilis* QST 713 per day (1 g; Baymix GROBIG, Bayer de Mexico).
- L-arginine group (n = 54): Basal diet + 20 g of L-arginine (purity ≥ 98.5%, CJ Biotech Co., Ltd., China).
- Combined group (n = 54): Basal diet + both 10^10^ CFU *B. subtilis* QST 713 and 20 g L-arginine.

Supplements were top-dressed onto the morning feed daily from day 85 of gestation until farrowing. The *B. subtilis* dosage followed protocols from previous studies by Khoudphaithoune *et al*. [25], and the L-arginine dosage aligned with earlier findings [17, 29]. The supplemented arginine levels exceeded National Research Council (NRC) requirements [31].

### Data collection and variable definitions

Farrowing dates were recorded to calculate gestation length. Total piglets born (TB) included live-born, stillborn, and mummified piglets. When farro-wing occurred during working hours, IBWs (in grams) were measured immediately using a digital hook scale (5 g precision; Weihang, China). For nighttime farrowings, birth weights were recorded within 6 h post-parturition.

- TBW (kg): Sum of IBWs per litter.
- Litter birth weight of born-alive piglets (NBABW, kg): Sum of weights of all live-born piglets.
- Within-litter variations (coefficient of variation in birth weight [CVBW], %): (Standard deviation/mean IBW) × 100.
- Runt piglets: Birth weight ≤1.1 kg [32].
- Large piglets: Birth weight >1.5 kg.

### Statistical analysis

Data were analyzed using the IBM Statistical Package for the Social Sciences (SPSS) Statistics version 22.0 (IBM Corp., NY, USA) and R program vers-ion 4.2.2 (The R Foundation, Vienna, Austria). Linear mixed models (LMMs) were used for continuous variables (IBW, TBW, NBABW, TB, number of piglets born alive [NBA], CVBW) and generalized LMMs (GLMMs) were used for binary outcomes (runt, large, stillbirth, mummification).

#### LMM for IBW

Y_ijk1_ = μ + A_i_ + B_j_ + AB_ij_ + C_k_ + S_1_ + e_ijk1_

Where:

- Y_ijk1_ = IBW
- A_i_ = Fixed effect of *B. subtilis* (presence/absence)
- B_j_ = Fixed effect of L-arginine (presence/absence)
- AB_ij_ = Interaction effect
- C_k_ = Random effect of farrowing batch
- S_1_ = Random effect of sow
- e_ijk1_ = Residual error.

#### LMM for TBW, NBABW, TB, NBA, CVBW

Y_ijk_ = μ + A_i_ + B_j_ + AB_ij_ + C_k_ + e_ijk_

Pairwise comparisons among treatments were performed using LMMs in SPSS. Batch and sow were included as random effects for IBW; only batch was included as a random effect for the other parameters. Model fit was assessed through residual histogram inspection.

#### GLMM for binary outcomes

logit (μ_ijk1_) = μ + A_i_ + B_j_ + AB_ij_ + C_k_ + S_1_ + e_ijk1_

Where:

- logit(μ_ijk1_) = Log-odds of the outcome
- A_i_, B_j_= Fixed effects of *B. subtilis* and L-arginine
- AB_ij_ = Interaction term
- C_k_, _1_ = Random effects (batch and sow)
- e_ijk1_ = Residual.

Pairwise comparisons for binary outcomes were conducted using the emmeans function in the emmeans package in R program version 4.2.2 (The R Foundation). Model adequacy for GLMMs was evaluated by checking dispersion parameters using Pearson residuals.

### Post hoc power analysis

A *post hoc* power analysis for the primary outcome (IBW) was conducted using LMMs, with treatment as the fixed effect and sow and batch as random effects. Simulations (n = 1,000) using the simr package in R program version 4.2.2 (The R Foundation) showed that the study had 90.6% power (95% confidence interval: 88.62%–92.34%) to detect the observed treatment effect at α = 0.05.

## RESULTS

### Overview of farrowing outcomes

A total of 3,364 piglets were born from the 247 sows enrolled in the study. Among these, 2,971 piglets (88.3%) were born alive, 253 (7.5%) were stillborn, and 140 (4.2%) were mummified.

### Effect on IBW

A marginal interaction between *B. subtilis* and L-arginine supplementation was observed for IBW of piglets (p = 0.084) (Table 2). Neither *B. subtilis* alone (1337.2 g vs. 1310.0 g, p = 0.387) nor L-arginine alone (1353.6 g vs. 1310.0 g, p = 0.082) significantly increased IBW compared to the control. However, co-supplementation significantly enhanced IBW by 124.7 g (1434.7 g vs. 1310.0 g, p < 0.001). IBW was also significantly higher in the co-supplemented group compared to the *B. subtilis* group (p < 0.0001) and the L-arginine group (p = 0.002).

**Table 2:** Effects of *B. subtilis* and L-arginine supplementation on reproductive parameters in sows.

Effect	Birth weight parameters				Litter size parameters		Birth outcomes			
		
	IBW (g)	TBW (kg)	NBABW (kg)	CVBW (%)	TB (piglets)	NBA (piglets)	Runt (%)	Large (%)	SB (%)	MM (%)
*B. subtilis* × L-arginine										
*No B. subtilis*										
No L-arginine	1310.0^a^	16.9^a^	15.9 ^a^	14.4	13.3	11.5	14.4^a^	19.5^a^	9.3	4.0
L-arginine	1353.6^a^	17.8^a^	16.4^a^	15.1	13.5	12.1	11.4 ^a^	24.7^a^	5.5	5.3
*B. subtilis*										
No L-arginine	1337.2^a^	18.1^a^	16.6^ab^	14.8	13.9	12	13.9^a^	25.6^a^	8.5	3.2
L-arginine	1434.7^b^	19.6^b^	18.1^b^	13.9	13.9	12.6	4.0^b^	33.7^b^	5.9	4.1
Main effect mean										
*B. subtilis*										
No	1326.9^x^	17.3^x^	16.1^x^	14.6	13.4	11.7	13.2^x^	21.5^x^	7.8	4.5
Yes	1386.2^y^	18.9^y^	17.4^y^	14.3	13.9	12.3	8.9^y^	29.7^y^	7.3	3.8
L.arginine										
No	1320.7^α^	17.4^α^	16.1	14.5	13.5	11.7	14.2^α^	21.9^α^	9	3.8
Yes	1394.6^ß^	18.7^ß^	17.3	14.5	13.7	12.3	7.6^ß^	29.3^ß^	5.8	4.7
*B. subtilis* × L-arginine Interaction (p-value)	0.084	0.329	0.244	0.672	0.72	0.963	0.008	0.31	0.714	0.949
SEM	4.365	0.253	0.271	0.451	0.169	0.214	0.006	0.008	0.005	0.003

IBW=Individual birth weight (g), TBW=Total litter birth weight (kg), NBABW=Litter birth weight of born-alive piglets, CVBW=Coefficient of variation in piglet birth weight (%), TB=Total born (piglets), NBA=Number of born-alive piglets (piglet), SB=Stillbirth (%), MM=Mummy (%), SEM=Standard error of the mean, *B. subtilis=Bacillus subtilis*. The values are presented as mean and the standard error of the mean which was calculated from the pooled values. Means in the same column with different superscripts (a, b), (x, y) or (α, ß) differed significantly (p < 0.05).

### Effect on runt piglet proportion

A significant interaction was observed between *B. subtilis* and L-arginine for the proportion of runt piglets (p = 0.008). *B. subtilis* alone (13.9% vs. 14.4%, p = 0.947) and L-arginine alone (11.4% vs. 14.4%, p = 0.469) did not significantly reduce runt piglet incidence. In contrast, co-supplementation significantly lowered the proportion of runt piglets to 4.0% compared to 14.4% in the control group (p < 0.001). This reduction was also significant compared to the *B. subtilis* (p = 0.0001) and L-arginine (p = 0.002) groups.

### Effect on total and born-alive litter weight

Supplementation with either *B. subtilis* or L-arginine alone did not result in significant changes in TBW or the litter birth NBABW (p > 0.05). However, co-supplementation significantly increased TBW (19.6 kg vs. 16.9 kg, p < 0.001) and NBABW (18.1 kg vs. 15.9 kg, p = 0.006) compared to the control. TBW and NBABW were also significantly higher in the co-supplemented group than in the *B. subtilis* group (TBW: p = 0.041; NBABW: p = 0.051) and the L-arginine group (TBW: p = 0.018; NBABW: p = 0.032). No significant interaction effect was detected for TBW or NBABW.

### Effect on the proportion of large piglets

Neither *B. subtilis* (25.6% vs. 19.5%, p = 0.541) nor L-arginine (24.7% vs. 19.5%, p = 0.658) alone significantly affected the proportion of large piglets (>1.5 kg). In contrast, co-supplementation led to a signi-ficant increase in large piglet proportion compared to the control group (33.7% vs. 19.5%, p = 0.0002), as well as when compared to *B. subtilis* (p = 0.048) and L-arginine (p = 0.026) alone. However, no significant interaction effect between *B. subtilis* and L-arginine was observed for this parameter.

### Effect on other reproductive parameters

No significant effects of *B. subtilis*, L-arginine, or their combination were observed for TB, NBA, CVBW, stillbirth rate, or mummification rate (p > 0.05 for all).

## DISCUSSION

To the best of our knowledge, this is the first study to evaluate the interactive effects of *B. subtilis* and L-arginine, co-supplemented from day 85 of gestation until farrowing, on reproductive performance in sows raised under medium-scale commercial farm conditions in Vietnam. The results clearly demonstrated that co-supplementation significantly increased piglet birth weight, raised the proportion of piglets weighing over 1.5 kg, and reduced the proportion of piglets weighing less than 1.1 kg. In addition, TBW and the birth weight of live-born piglets (NBABW) were significantly improved. These enhancements were driven by a synergistic interaction between *B. subtilis* and L-arginine, as neither supplement alone produced comparable effects.

### Mechanistic basis for synergistic effects

The observed synergistic effect may be attributed to the complementary physiological roles of *B. subtilis* and L-arginine. *B. subtilis* QST 713 is known to prod-uce antimicrobial compounds, such as bacillaene, diffi-cidin, macrolactin, and ericin, and the biosurfactant surfactin [33], which exhibit inhibitory activity against a range of animal pathogens, including *Campylobacter jejuni*, *Staphylococcus aureus*, *Enterococcus* spp., *Pseudomonas aeruginosa*, *Clostridium difficile*, *Bact-eroides* spp., and *E. coli* [34–36]. These properties likely contribute to improved gut microbiota health. Furth-ermore, *B. subtilis* produces enzymes such as amylase, cellulase, and protease that enhance nutrient digestion and absorption [28].

L-arginine, on the other hand, functions primarily through the nitric oxide and polyamine pathways [37], promoting angiogenesis and improving placental blood flow [30], which in turn supports placental growth [29] and enhances nutrient and oxygen delivery to the fetus. Thus, while *B. subtilis* may improve overall nutrient availability through gut health, L-arginine enhances the efficiency of nutrient transfer to the fetus. Together, these mechanisms likely contributed to the observed improvements in fetal development and birth weight.

### Comparison with previous studies

The increase in IBW observed in the co-supplem-ented group is consistent with previous findings. A 70 g increase in birth weight was reported with *B. subtilis* supplementation alone [26], while supplementation with both *B. subtilis* and *Bacillus amyloliquefaciens* yielded similar improvements [38, 39]. For L-arginine, one study reported a 100 g increase in piglet birth weight with supplementation during the last month of gestation [17]. Our findings suggest that the combination of *B. subtilis* and L-arginine amplified the benefits observed in these individual trials.

### Impact on litter weight outcomes

Co-supplementation significantly increased TBW and NBABW. These results align with some reports on the beneficial effects of probiotic or L-arginine supplem-entation [18, 23, 40–42], although other studies have reported no such improvement [13–15, 17, 43]. In some cases, increased TBW/NBABW occurred alongside higher litter sizes but lower IBW [23, 42], while in others, both litter size and IBW increased numerically [18, 40, 41]. In the present study, the observed increase in TBW and NBABW was primarily due to increased fetal growth, as TB and NBA remained relatively unchanged across treatments. This reinforces the conclusion that co-supp-lementation enhanced fetal development without signi-ficantly affecting litter size.

### Reduction in runt piglets and increase in large piglets

A notable outcome was the significant reduct-ion in the proportion of runt piglets (<1.1 kg) and a simultaneous increase in the proportion of large piglets (>1.5 kg). Previous studies on L-arginine sup-plementation have yielded mixed results: 0.79% L-arginine had no effect on piglets <1.2 kg or >1.6 kg, and 0.28% L-arginine unexpectedly increased the proportion of lighter piglets [15]. Other studies using 1% L-arginine [13] or 25.5 g/day [19] found no significant effects on the incidence of piglets <1 kg or >1.7 kg. However, a 0.5% L-arginine dose did increase the proportion of piglets above 1.35 kg [17]. The reduction in runt piglets and increase in heavier piglets observed in our study are likely to translate into reduced perinatal and preweaning mortality, as well as improved growth performance in later stages [3, 44].

### No significant effect on CVBW, stillbirth, or mummification

Despite improvements in birth weight, co-supplem-entation did not significantly affect CVBW, stillbirth rate, or mummification rate. These results are consistent with several prior studies that reported no effect of L-arginine or probiotics on CVBW [14–15, 17, 25, 45]. Only one study to date has documented a reduction in CVBW following L-arginine supplementation, though it was not accompanied by an increase in birth weight [19]. Similarly, while some studies have observed a decrease in stillbirths with L-arginine or *B. subtilis* supplementation [18, 25, 26], our results did not confirm this. As birth weight only modestly explains variation in CVBW and stillbirth [1, 32], the absence of significant change in these parameters is plausible.

### Limitations and future directions

Although the study demonstrated significant improvements in birth outcomes with co-supplem-entation, it did not explore the biological mechanisms underlying these effects. Future research should include assessments of placental angiogenesis, fetal muscle and fat development, and changes in the maternal microbiome to better understand the physiological pathways involved. Molecular, histological, and micro-biome-based analyses are warranted to confirm the proposed mechanisms and refine supplementation strategies further.

## CONCLUSION

This study provides the first evidence of a synergistic effect between *B. subtilis* QST 713 and L-arginine supplementation during late gestation on improving reproductive performance in Landrace × Yorkshire sows under commercial farming conditions in Vietnam. Co-supplementation from day 85 of gestation until farrowing resulted in a significant increase in IBW (by 124.7 g), TBW (19.6 kg vs. 16.9 kg), and birth weight of live-born piglets (18.1 kg vs. 15.9 kg). It also substantially reduced the proportion of runt piglets (4.0% vs. 14.4%) and increased the proportion of large piglets (>1.5 kg) from 19.5% to 33.7%. These improvements were not observed with either supplement alone, underscoring the importance of their interactive effects.

The findings suggest that targeted co-supplem-entation of *B. subtilis* and L-arginine is a practical and economically viable strategy to enhance piglet viability and early-life growth potential. By shifting the birth weight distribution toward heavier piglets, this approach may reduce perinatal mortality, improve postnatal performance, and contribute to overall productivity gains in commercial swine herds.

A major strength of this study lies in its randomized design and execution under real-world, medium-scale commercial conditions, enhancing the relevance and applicability of the findings. The factorial approach enabled a detailed assessment of both the individual and combined effects of the interventions.

In conclusion, this study supports the implem-entation of dietary co-supplementation with *B. subtilis* and L-arginine as a strategic nutritional intervention to improve reproductive efficiency and piglet quality in modern swine production. Future studies are encou-raged to investigate the long-term effects on piglet growth and survival, and to elucidate the underlying biological mechanisms through molecular and microb-iome-focused approaches.

## DATA AVAILABILITY

All the generated data are included in the manuscript.

## AUTHORS’ CONTRIBUTIONS

TK, NHN, DTKL, NVT, BVD, and PS: Conceived and designed the study. TK and NHN: Collected the data and drafted and revised the manuscript. TK, NHN, and PS: Analyzed the data and interpreted the results. All the authors participated in scientific discussion and read and approved the final manuscript.
